# Management Dilemma in a Large Fungating Squamous Cell Carcinoma Over a Marjolin’s Ulcer on the Chest Wall Coexisting With Severe Post-Burn Contracture of the Neck

**DOI:** 10.7759/cureus.42321

**Published:** 2023-07-23

**Authors:** Suruchi Sanjay, Aman Choudhary, Meena Kumari, Bharati Pandya

**Affiliations:** 1 General Surgery, All India Institute of Medical Sciences, Bhopal, Bhopal, IND

**Keywords:** premalignant condition, post-burn contracture, infiltrating local anesthesia, fiberoptic guided endotracheal intubation, wide local excision, marjolin's ulcer

## Abstract

Marjolin’s ulcer is a premalignant condition occurring over scars and chronically inflamed tissue. Squamous cell carcinoma is the most common malignancy associated with Marjolin’s ulcer. The progression of disease and metastasis is relatively slow due to scarring and meagre lymphatic and vascular supply over these scars. We present a case of a large fungating squamous cell carcinoma in a patent having a burn scar over his chest as well as having post-burn contracture of the neck, posing problems in management due to intubation issues. We managed him unconventionally with serial excision under local anesthesia and skin grafting of the wound.

## Introduction

Marjolin’s ulcer was first described by Dr Jean-Nicolas Marjolin in the year 1828 [1]. It arises due to chronic inflammatory reactions in wounds that failed to heal. Around 2% of thermal burns scars and 0.7% of osteomyelitic lesions develop into Marjolin's ulcers [2]. Squamous cell carcinoma is the most common malignance arising in a Marjolin’s ulcer. Severe neck contracture, again a common sequela of burns, can be a challenging condition to manage, particularly in a patient requiring emergency or similar surgery, and requires serious consideration. Ideally, surgical intervention is necessary to release the contracture; it may not be feasible in the context of surgeries that cannot wait, especially in patients who also have other comorbidities. A careful balance between the risks and benefits has to be taken into account.

## Case presentation

**Figure 1:**
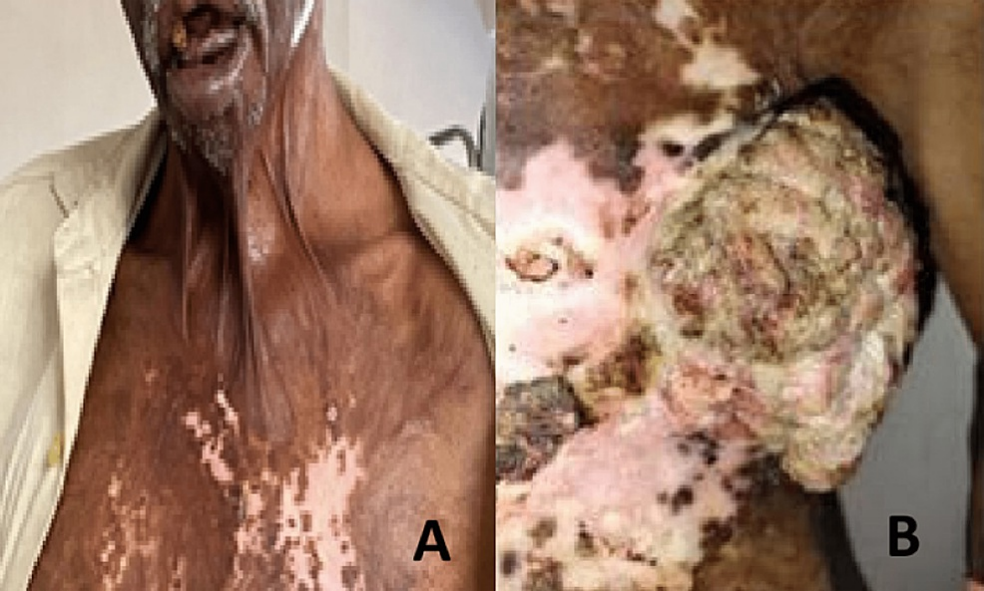
The patient had (A) severe post-burn neck contraction with eversion of the lower lip and (B) a 15 cm x 10 cm fungating mass over his scarred left chest wall. The patient in the profile with post-burn neck contracture and the mass.

A 62-year-old male, farmer by occupation, with a history of burn injury over the neck and left chest region during childhood presented with a huge mass over the left chest wall to the outpatient department. He had burns sustained at 10 years of age, followed by burn contractures over the neck and left axilla, and an unstable scar over the chest wall, for which he kept taking local treatment. He developed a gradually progressive mass over the left chest wall four months prior to presentation (Figure 1), which started bleeding off and on. Subsequently, it also started exuding foul pus and necrotic material, for which he presented to the outpatient department. Dressing of the mass at his rural center was getting increasingly painful due to its size, bleeding, and necrosis, which was accompanied by episodes of fainting. He had no history of substance abuse. On examination, the patient was pale and emaciated, and had a severe neck contracture with ectropion of the lower lip. He had a 15 x 10 cm fungating mass, with foul-smelling pus discharge involving the scarred left chest wall close to the left axilla. (Figures 1A, 1B).

On investigation, the patient had a hemoglobin level of 7 g%, which was corrected with blood transfusions, and other blood investigations were within normal limits. Contrast-enhanced CT of the thorax was suggestive of a soft tissue mass, of likely neoplastic etiology, but not infiltrating the muscles or ribs. There were no distant metastases found on investigations. Biopsy confirmed squamous cell carcinoma.

**Figure 2:**
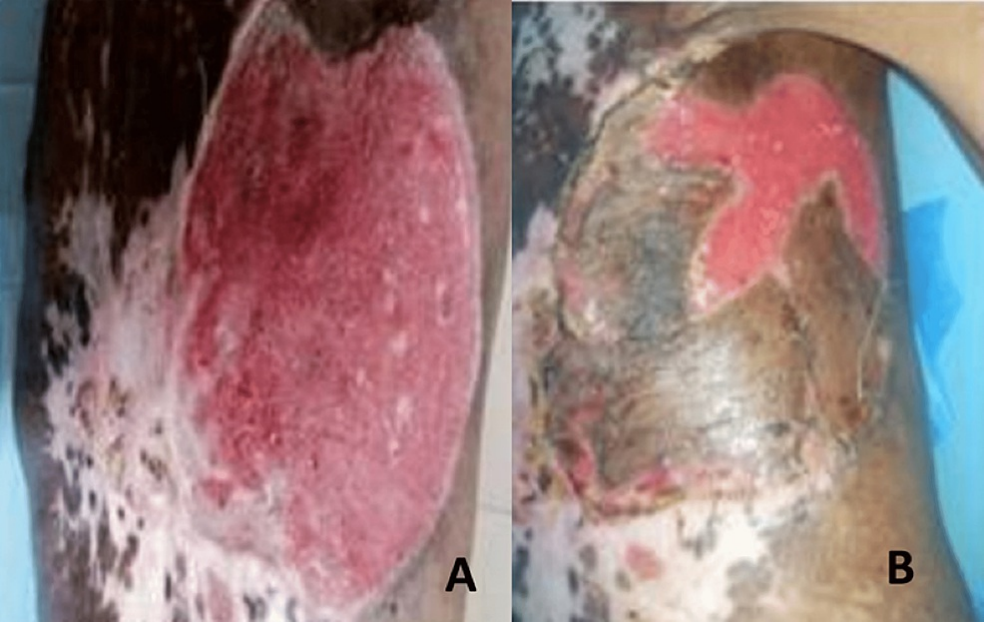
The raw area (A) after complete resection of the tumor, with negative pathological margins for tumor, and (B) after being partially grafted with a split-thickness skin graft. The raw area extended from 5 cm lateral of the left sternal border to the posterior axillary fold horizontally. Vertically, the extent was from the second rib to the ninth rib below. Complete resection and healthy bed for skin grafting.

The patient was ASA grade 3 (chronic obstructive pulmonary disease), with burn contracture over the neck (difficult intubation). This made him unfit for general anesthesia. Hence, the patient was planned under local anesthesia and sedation and anesthesia backup, which was not actually required. The patient had a very low body weight of 29 kg only, and hence adequate anesthesia for a single-stage excision was not possible. The mass was excised in three sittings, with removal of the superficial part first, followed by anterior and deep margins subsequently, over three consecutive days. The local anesthetic used was lidocaine 1% with adrenaline (infiltration lignocaine concentration in solution is 1 g per 100 mL or 10 mg/mL). The maximum permissible dose is of 7 mg/kg of body weight. (For a patient weight 35 kg, the permissible maximum was 245 mg or a total of 24.5 mL per sitting). Area to be infiltrated measured 10 cm x 15 cm, and hence three sittings were needed. The needle direction with infiltration was initially parallel to the edge followed by a fan-shaped fashion to cover the resection area mapped per sitting. Split-thickness skin grafting was performed afterward over the healthy raw area, again under local anesthesia in two sittings, with the graft being harvested from the patient's thigh (Figures 2A, 2B).

**Figure 3:**
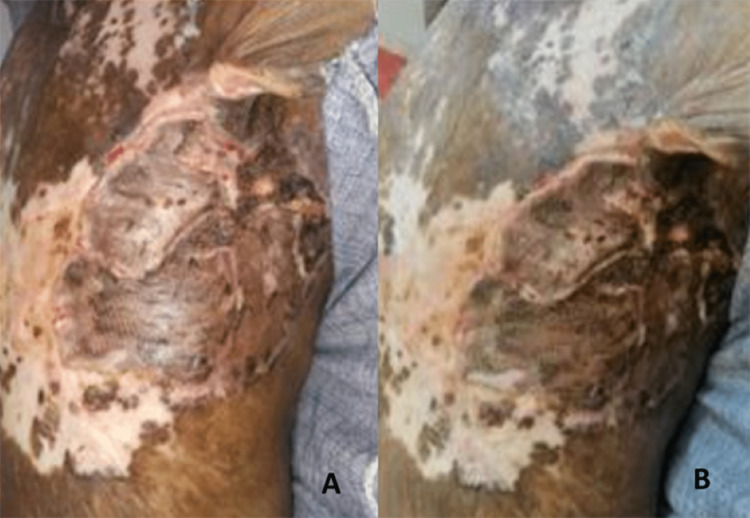
Completely grafted area (A) just before discharge and (B) after six weeks of follow-up, total three months after resection of tumor. The whole area was grafted before the patient was discharged, as he came from a remote rural area.

Once the healthy granulation tissue appeared and the histopathology confirmed the base to be free of tumor, split skin grafting was performed in two sittings (above) to cover the entire raw area. The wound was healthy in the postoperative period (Figure 3A), even after six weeks of follow-up (Figure 3B).

Histopathology showed moderately differentiated squamous cell carcinoma. Its excised base had dense inflammatory infiltrates, and the deep margin revealed fibro-muscular tissue and congested blood vessels; there was no malignant spread in these tissue planes. Since there was no local or distant spread, the patient was kept on general follow-up and has been asymptomatic for the past one year. He is now due for release of his neck contracture. His family has not been willing to undergo a surgery, which he has not had over years. The tumor was treated because of its emergent nature. There were no suspicious lesions or signs of recurrence in the follow-up visits.

## Discussion

Marjolin’s ulcer is a non-healing, pre-malignant, chronically inflamed ulcer that forms over scar tissue. It is sometimes an aggressive cutaneous malignancy associated with chronic wounds, venous stasis ulcers, lupus vulgaris, pressure sores, osteomyelitis, anal fistulae, pilonidal abscesses, and radiotherapy [1,2]. It occurs on an average around 30 years after an injury to the skin [3]. It has a potential to spread locally, and also distant metastasis may occur if neglected and untreated. A wide margin of clearance gives adequate protection against recurrence. Our patient’s lung status and neck contracture were unfavorable for general or regional anesthesia. Therefore, excision was performed from the superficial to deep planes, in stages. Table 1 describes the differences between de novo squamous cell carcinoma and that arising in a Marjolin's ulcer, which, as can be seen, requires a wider margin, as described by Iqbal et al. [1]. In-depth spread is slow and less compared to circumferential spread (Table 1).

**Table 1:** Differences in a squamous cell carcinoma arising de novo and that with Marjolin’s ulcer.

Features	Squamous cell carcinoma in Marjolin’s ulcer	De novo squamous cell carcinoma
Sex ratio	3:1	1.1–1.7:1
Average age of presentation	52 years	66 years
Common sites for presentation	Lower extremities, scalp (+ any area with scarring and non-healing ulcer)	Head and neck
Excision margins	2–4 cm	4–6 mm
Metastatic rates	27.5–40 %	3–23%

Recent advances that need further randomized controlled trials and widespread studies for early diagnosis of squamous cell carcinoma have come up. Tsai et al. way back in 2009 evaluated the use of a non-invasive imaging technique called optical coherence tomography (OCT) for diagnosing and monitoring squamous cell carcinoma [4]. The authors found that OCT was able to detect early changes indicative of cancer, suggesting that it may be a useful tool for detecting squamous cell carcinoma in its early stages [4]. Squamous cell carcinoma in situ is an intra-epidermal malignancy of the skin with potential to progress to invasive carcinoma. Commonly used treatments are surgical excision, cryotherapy, photodynamic therapy, laser ablation, curettage with cautery, radiotherapy, topical 5-fluorouracil, and topical imiquimod [5,6]. Brachytherapy is a form of radiotherapy used for squamous cell carcinoma, in particular localizations, and serves as a valuable tool to deliver exact radiation depot within the tumor mass. It can be used for skull skin, face, inoperable tumors, and relapses following surgery. It is usually not suitable for invasive treatment. The complication rate is acceptable and treatment costs are low [7]. Post-burn contracture of the neck poses a difficult airway challenge to the anesthesiologist. The anesthesiologist needs a pre-planned strategy for the difficult airway. Choices available are awake fiberoptic-guided intubation, use of laryngeal mask airway, or endotracheal intubation, along with avoidance of neuromuscular blocking agents. This could be followed by pre-induction release of the neck scar under local anesthesia and sedation [8,9]. This next has to be followed by direct laryngoscopy and intubation or even video-laryngoscope guided intubation. The bottom line remains that increasing awareness among the general population and motivating them for early presentation for treatment will go a long way in preventing morbidity and mortality.

## Conclusions

There should be a high index of suspicion for recent changes in long-standing chronic unstable scars, ulcers, and sinuses, as early diagnosis through clinical suspicion and biopsy can result in quick and appropriate treatment, leading to a more favorable prognosis. The presence of any non-healing ulcer needs management with excision and skin grafting before it undergoes neoplastic transformation. Similarly, any neck contracture needs correction at the earliest because in emergencies where intubation may be required, airway maintenance can cause severe hindrance, as in our situation, where removal of the tumor could not wait due to bleeding, infection, and malignancy. This led to an urgent scenario adding to dilemmas in management, which could have resulted in serious morbidity. In rural India, this issue in management needs tackling with increasing awareness toward early management.
